# Pharmacological regimens for eradication of Helicobacter pylori: an overview of systematic reviews and network meta-analysis

**DOI:** 10.1186/s12876-016-0491-7

**Published:** 2016-07-26

**Authors:** Yiqiao Xin, Jan Manson, Lindsay Govan, Robin Harbour, Jenny Bennison, Eleanor Watson, Olivia Wu

**Affiliations:** 1 Health Economics and Health Technology Assessment (HEHTA), Institute of Health and Wellbeing, University of Glasgow, Glasgow, UK; 2 Knowledge and Information, Healthcare Improvement Scotland, Glasgow, UK; 3 Scottish Intercollegiate Guideline Network (SIGN), NHS Education for Scotland, Royal College of General Practitioners (Scotland), Mill Lane Surgery, Edinburgh, UK; 4 Department of gastroenterology, Royal Infirmary of Edinburgh, NHS Lothian, Edinburgh, UK

**Keywords:** Helicobacter pylori, Eradication, Systematic review, Network meta-analysis

## Abstract

**Background:**

Approximately half of the world’s population is infected with Helicobacter pylori (H.pylori), a bacterium shown to be linked with a series of gastrointestinal diseases. A growing number of systematic reviews (SRs) have been published comparing the effectiveness of different treatments for H.pylori infection but have not reached a consistent conclusion. The objective of this study is to provide an overview of SRs of pharmacological therapies for the eradication of H.pylori.

**Methods:**

Major electronic databases were searched to identify relevant SRs published between 2002 and February 2016. Studies were considered eligible if they included RCTs comparing different pharmacological regimens for treating patients diagnosed as H.pylori infected and pooled the eradication rates in a meta-analysis. A modified version of the ‘A Measurement Tool to Assess Systematic Reviews’ (AMSTAR) was used to assess the methodological quality. A Bayesian random effects network meta-analysis (NMA) was conducted to compare the different proton pump inhibitors (PPI) within triple therapy.

**Results:**

30 SRs with pairwise meta-analysis were included. In triple therapy, the NMA ranked the esomeprazole to be the most effective PPI, followed by rabeprazole, while no difference was observed among the three old generations of PPI for the eradication of H.pylori. When comparing triple and bismuth-based therapy, the relative effectiveness appeared to be dependent on the choice of antibiotics within the triple therapy; moxifloxacin or levofloxacin-based triple therapy were both associated with greater effectiveness than bismuth-based therapy as a second-line treatment, while bismuth-based therapy achieved similar or greater eradication rate compared to clarithromycin-based therapy. Inconsistent findings were reported regarding the use of levofloxacin/moxifloxacin in the first-line treatment; this could be due to the varied resistant rate to different antibiotics across regions and populations. Critical appraisal showed a low-moderate level of overall methodological quality of included studies.

**Conclusions:**

Our analysis suggests that the new generation of PPIs and use of moxifloxacin or levofloxacin within triple therapy as second-line treatment were associated with greater effectiveness. Given the varied antibiotic resistant rate across regions, the appropriateness of pooling results together in meta-analysis should be carefully considered and the recommendation of the choice of antibiotics should be localized.

**Electronic supplementary material:**

The online version of this article (doi:10.1186/s12876-016-0491-7) contains supplementary material, which is available to authorized users.

## Background

Helicobacter pylori (H.pylori) is one of the most common human infections with a worldwide prevalence of approximately 50 %. In the United States (US) and Europe, the prevalence of H.pylori is estimated to be 20 % to 50 %, varying in different socioeconomic, age and ethnic groups and geography [1, 2]. In developing countries, the prevalence has been reported to be as high as 70 % [3]. H.pylori is usually latent and asymptomatic; however, increasing evidence has demonstrated the link between H.pylori infection and the pathogenesis of a series of upper gastrointestinal diseases: functional dyspepsia, chronic gastritis, peptic ulcer disease, gastric cancer and gastric mucosa-associated lymphoid-tissue lymphoma [4–9].

Eradication of H.pylori has been shown to be associated with increased rate of peptic ulcer healing and reduced risk of gastric cancer [10, 11]. Standard triple therapy, which includes a proton pump inhibitor (PPI), clarithromycin, and amoxicillin or metronidazole, is recommended as first-line eradication therapy for H.pylori infection in clinical guidelines worldwide [12–15]. A treatment alternative also widely recommended is bismuth-based quadruple therapy, which contains a PPI or H_2_ receptor antagonist (H_2_RA), bismuth, metronidazole, and tetracycline. Other treatment options include varying individual drugs within the triple therapy and quadruple therapy based regimens. More recently, sequential therapy of these multiple treatment options also has been introduced. In the US, the American College of Gastroenterology guideline (2007) recommends clarithromycin-based triple therapy for first-line eradication in patients who have not previously been treated with clarithromycin and are not allergic to penicillin. For patients who are allergic to penicillin or have previously received a macrolide antibiotic, a bismuth quadruple therapy is preferred [14].

Although these recommendations specified the type of antibiotics in the regimen, the choice of PPIs was not specified. Based on the available evidence at the time when the guidelines were produced, the relative effectiveness of PPIs was assumed to be comparable. Furthermore, in recent years, a decline in the effectiveness of the treatment regimens has been observed due to increasing clarithromycin resistance; this may have an impact on the relative effectiveness of these treatment strategies [16]. A 12-year retrospective study published in 2008 showed that the eradication rate of standard therapy decreased from 90.6 % in 1997 to 74.8 % [17].

In the past decade, several systematic reviews have evaluated the effectiveness of individual specific pharmacological regimens for H.pylori eradication. These reviews compared the eradication rate by different PPIs and antibiotics, triple versus quadruple therapy, or PPI versus H_2_RA, but the conclusions of these reviews were not always consistent.

The Scottish Intercollegiate Guidelines Network (SIGN) published their recommendations on H.pylori eradication in the dyspepsia guideline in 2003 and is due to update their guidance [18]. This study aims to systematically evaluate the current evidence (since 2003) on the effectiveness of H.pylori eradication therapies for the patients diagnosed as H.pylori infection through an overview of systematic reviews.

## Methods

An overview of systematic reviews was carried out according to the general principles of systematic reviewing methodology [19]. A Bayesian network meta-analysis (NMA) was conducted to compare the eradication rates by using different PPIs within triple therapy.

### Eligibility criteria

All systematic reviews comparing different drug therapies for the eradication of H.pylori infection that fulfilled the following criteria were included:

- **Patient** — studies of adult patients who were naïve to treatment (first-line therapy) or have previous treatment failures (second-line therapy).
- **Intervention/Comparator** — studies comparing any pharmacological regimens.
- **Outcome measure** — studies reporting pooled eradication rates measured by urea breathe testing or gastric mucosal biopsy four weeks after completion of treatment, as the primary outcome. Secondary outcome measures may include adverse events rates and rates of discontinuation of therapy due to severe adverse events.
- **Design** — systematic reviews and meta-analyses of data from randomized controlled trials (RCTs).

### Exclusion criteria

Studies were excluded if they focused on comparing the variation of dose or duration of the same drug combination; if no meta-analysis was conducted; meta-analysis included observational studies; or the included RCTs in the meta-analysis were not clearly specified. Conference abstracts were excluded due to lack of details for data extraction and quality assessment. Studies on furazolidone were excluded because it is no longer available in the US and the United Kingdom (UK) due to severe side effects. No language exclusions were applied. As this work was initiated by the SIGN guideline update, studies published prior to 2002 were excluded.

### Search strategy

Four major electronic databases were searched: MEDLINE, EMBASE, the Cochrane Library and the Database of abstracts of review of effects. Relevant keywords were used to develop appropriate search strategies; these are shown in the Additional file 1. The primary search was carried out in November 2012 and updated in March 2016.

### Study selection

Two reviewers (JM and YX) independently reviewed the titles and abstracts of all retrieved studies for identification of potentially relevant systematic reviews. After the initial screening, the full texts of studies deemed relevant were obtained and reviewed in detail. The discrepancy was addressed by discussion or a third reviewer (OW). Reference list of included studies was also checked to identify any potentially relevant studies that may not have been identified by the electronic searching.

### Data extraction

For each included systematic review, the following data were extracted by two reviewers independently: first author, publication year and country; objective; search database and selection criteria; number of included studies in the review and meta-analysis; number of patients in the meta-analysis; patient characteristics; intervention and comparison; outcomes including eradication rate, adverse events rate, therapy discontinuation rate. In addition, as the resistant rate to antibiotics differs across regions, the country of the RCTs included in the meta-analysis was also extracted when the focus of the comparison was involved with antibiotics. To conduct the NMA, we also extracted data from the individual RCTs in the included systematic reviews, including: interventions in comparison, the total number of people in each arm and the number of people of which H.pylori had been eradicated.

### Quality assessment

To assess methodological quality of the included systematic reviews, a modified version of the ‘A Measurement Tool to Assess Systematic Reviews’ (AMSTAR) checklist [20] was used by two reviewers (JM and YX) independently to examine the following 11 aspects: (1) clearly defined research question; (2) study selection and data extraction carried out by two independent reviewers; (3) comprehensive literature search; (4) clear selection criteria; (5) list of included and excluded studies; (6) study characteristics appropriately extracted; (7) quality assessment documented; (8) results of quality assessment appropriately considered in reaching conclusions; (9) results combined appropriately; (10) publication bias assessed; (11) conflicts of interest declared. Studies were graded as “high quality (++)”, “acceptable (+)” or “low quality (0)”, based on the overall risk of bias and the likelihood that results may be changed by further research.

### Network meta-analysis (NMA)

A Bayesian random effect NMA was conducted to compare and rank all the PPIs within the triple therapy based on the eradication rates. When more than two interventions are being evaluated, conventional pairwise meta-analysis is limited in that it requires direct head-to-head evidence between interventions. In contrast, NMA allows the estimation of relative effects between multiple alternative interventions by incorporating both direct and indirect evidence [21, 22]. The NMA model used in this study is shown in Additional file 2. The odds ratios (ORs) for all pairwise comparisons of each treatment were calculated and presented in an interval plot. The median of the posterior distribution along with 95 % credible intervals (95%CrI) was reported. In addition, the PPIs were ranked based on their probability to be considered the best for the outcome of eradication rate of H.pylori.

Two sets of vague priors, uniform and inverse Gamma, were used for the Bayesian model, which were burned-in for 27,000 and 8,000 Markov Chain Monte Carlo iterations respectively until the convergence was met based on the Gelman-Rubin-Brooke statistic (within 1+/− 0.05). A further approximately 40,000 iterations were run until the MC error became lower than 5 % standard error and the results became stable. The median of the posterior distribution and credible intervals for ORs was reported. The analysis was performed using WinBUGS 1.4.3 [23].

## Results

### Results of search and selection

**Fig. 1:**
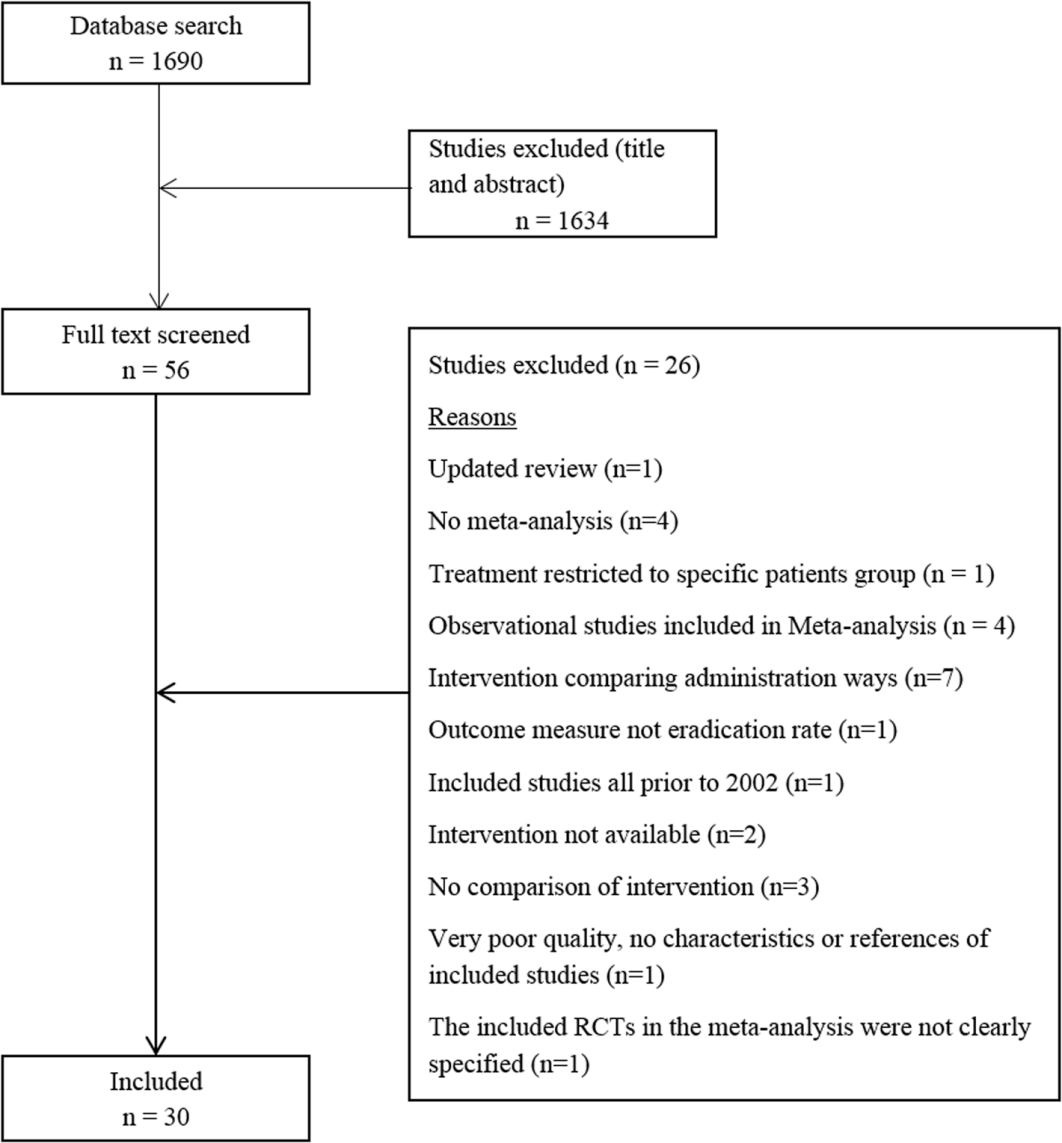
Flowchart showing the process of selecting systematic reviews on effectiveness of Helicobacter pylori eradication based on eligibility criteria

The search identified 1690 studies, of which 30 studies were included in this overview of systematic reviews. The flowchart of the screening process is shown in Fig. 1. The excluded studies at full-text screening stage are listed in Additional file 3: Table S1 with reasons for exclusion.

### Systematic reviews included in analysis

All the included studies were published between 2002 and 2015 in English, with the exception of three studies, which were published in Chinese [24–26]. Six studies exclusively evaluated second-line treatment for patients with at least one prior course of treatment failure [27–32]; 12 studies focused on treatment naive patients [25, 26, 33–42]; the remaining systematic reviews included RCTs for both first-line and second-line treatment. 13 studies evaluated treatments in patients with comorbid gastric diseases including peptic ulcer disease, duodenal ulcer, functional dyspepsia, chronic gastritis or other non-ulcer diseases [24, 27, 37–40, 43–49] (the remaining studies did not provide such data). In addition to the eradication rate, 15 systematic reviews also compared adverse events rates [25, 28–38, 41, 47, 50] and six compared the discontinuity rate (compliance rate) [25, 29–31, 37, 50]. The pooled eradication rates of different regimens in all of the included systematic reviews ranged between 47 % (data from three RCTs relating to standard triple therapy [30]) and 94 % (data from one RCT relating to esomeprazole-based triple therapy [46]) by intention to treat (ITT) analysis.

Based on the treatment regimens under comparison, the included studies were classified into the following five categories:

- Triple therapy with different PPIs
- Triple therapy with different antibiotics
- Triple therapy versus bismuth-based therapy
- PPI versus H_2_RA in triple therapy
- Other drug therapies

### Triple therapy with different PPIs

Seven studies evaluated the impact of different PPIs within a triple therapy regimen on H.pylori eradication rate (Table 1) [24, 42–46, 51]. These included both new (esomeprazole, rabeprazole) and older generations of PPIs (omeprazole, pantoprazole, lansoprazole); overall, the results were mixed, but a time trend was observed that studies published from 2006 onwards [24, 42, 46] suggested consistently that new generation of PPIs achieved greater eradication rate than the older generations. Amongst the new PPIs, the reported eradication rates ranged from 77 % (data from nine RCTs relating to rabeprazole-based triple therapy [43]) to 94 % (data from one RCT relating to esomeprazole-based triple therapy [46]); for the older generation PPIs, the reported eradication rates ranged from 75 % (data from four RCTs relating to omeprazole-based triple therapy [51]) to 88 % (data from two RCTs relating to omeprazole-based triple therapy [51]). Five studies compared esomeprazole with older generation PPIs in the triple therapy, of which, three reported a statistically significant benefit of esomeprazole in H.pylori eradication with OR of approximately 1.3 [24, 42, 46]. A similar effect was reported in one of the three studies comparing the effectiveness of the rabeprazole with the older generation PPIs (OR 1.21; 95%CI 1.02–1.42) [42]. Only one study compared the effectiveness of esomeprazole with rabeprazole and found no difference in eradication rate (OR 0.90; 95%CI 0.70–1.17) [42]. Similarly, no difference was observed when comparing within older generation PPIs [45, 51].

**Table 1:** Characteristics of systematic reviews comparing triple therapy with different PPIs (*n* = 7)

Author, year, country	Last search date	Disease	Intervention^c^	Comparator^c^	No. of studies in MA	No. of patients in MA	Eradication rates	Eradication rates odds ratio (95 % CI) by ITT	Quality assessment^b^
Gisbert et al. 2003-r Spain [43]	Sep 2002	HP infection; PUD/NUD/not reported	Rabeprazole	Omeprazole/Lansoprazole	12	2226	79 % vs. 77 %	1.15 (0.93–1.42)	+
			Rabeprazole	Omeprazole	9	1475	77 % vs. 77 %	1.03 (0.81–1.32)	
			Rabeprazole	Lansoprazole	7	1095	82 % vs. 79 %	1.20 (0.87–1.64)	
Vergara et al. 2003 Spain [51]	Sep 2002	HP infection	Omeprazole	Lansoprazole	4	1085	74.7 % vs. 76 %;	0.91 (0.69–1.21)^a^	+
			Omeprazole	Rabeprazole	4	825	77.9 % vs. 81.2 %	0.81 (0.58–1.15)^a^	
			Omeprazole	Esomeprazole	2	833	87.7 % vs. 89 %	0.89 (0.58–1.35)^a^	
			Lansoprazole	Rabeprazole	3	550	81 % vs. 85.7 %	0.77 (0.48–1.22)^a^	
Gisbert et al. 2004 Spain [44]	Jun 2003	HP infection; PUD +/−NUD	Esomeprazole	Omeprazole	4	1292	85 % vs. 82 %	1.19 (0.81–1.74)	+
Gisbert et al. 2004 Spain [45]	Sep 2002	HP infection; PUD +/−NUD	Pantoprazole	Omeprazole/Lansoprazole	7	1137	83 % vs. 81 %	1.00 (0.61–1.64)	+
			Pantoprazole	Omeprazole	1	974	83 % vs. 82 %	0.91 (0.49–1.69)	
			Pantoprazole	Lansoprazole	2	258	78 % vs. 75 %	1.22 (0.68–2.17)	
Wang et al. 2006 China [24]	Jul 2006	HP infection; DU, NUD, PUD	Esomeprazole	Omeprazole	11	2048	85.6 % vs. 81.6 %	1.30 (1.02–1.65)	0
Wang X et al. 2006 China [46]	2000–2005 (published date)	HP infection; PUD/NUD	Esomeprazole	Omeprazole/Pantoprazole	11	2146	86 % vs. 81 %	1.39 (1.09–1.75)	0
			Esomeprazole	Omeprazole	10	1946	85 % vs. 82 %	1.29 (1.01–1.65)	
			Esomeprazole	Pantoprazole	1	200	94 % vs. 82 %	3.44 (1.30–9.07)	
McNicholl et al. 2012 Spain [42]	Oct 2011	HP infection; naïve to therapy	Rabeprazole	Omeprazole/Lansoprazole/pantoprazole	21	2945	80.5 % vs. 76.2 %	1.21 (1.02–1.42)	0
			Esomeprazole	Omeprazole/Lansoprazole/pantoprazole	12	2598	82.3 % vs. 77.6 %	1.32 (1.01–1.73)	
			Rabeprazole	Esomeprazole	5	1574	76.7 % vs. 78.7 %	0.90 (0.70–1.17)	

*HP* H.pylori, *PPI* proton pump inhibitor, *PUD* peptic ulcer disease, *NUD* non-ulcer dyspepsia, *MA* meta-analysis, *ITT* intention to treat, *CI* confidence interval

^a^ Peto OR is reported here

^b^ Quality assessment: high quality (++): majority of criteria met, little or no risk of bias and results unlikely to be changed by further research. Acceptable (+): most criteria met, some flaws in the study with an associated risk of bias and conclusions may change in the light of further studies. Low quality (0): either most criteria not met or significant flaws relating to key aspects of study design, and conclusions likely to change in the light of further studies

^c^ The antibiotics are the same type and same dose for each arm of the RCTs

**Fig. 2:**
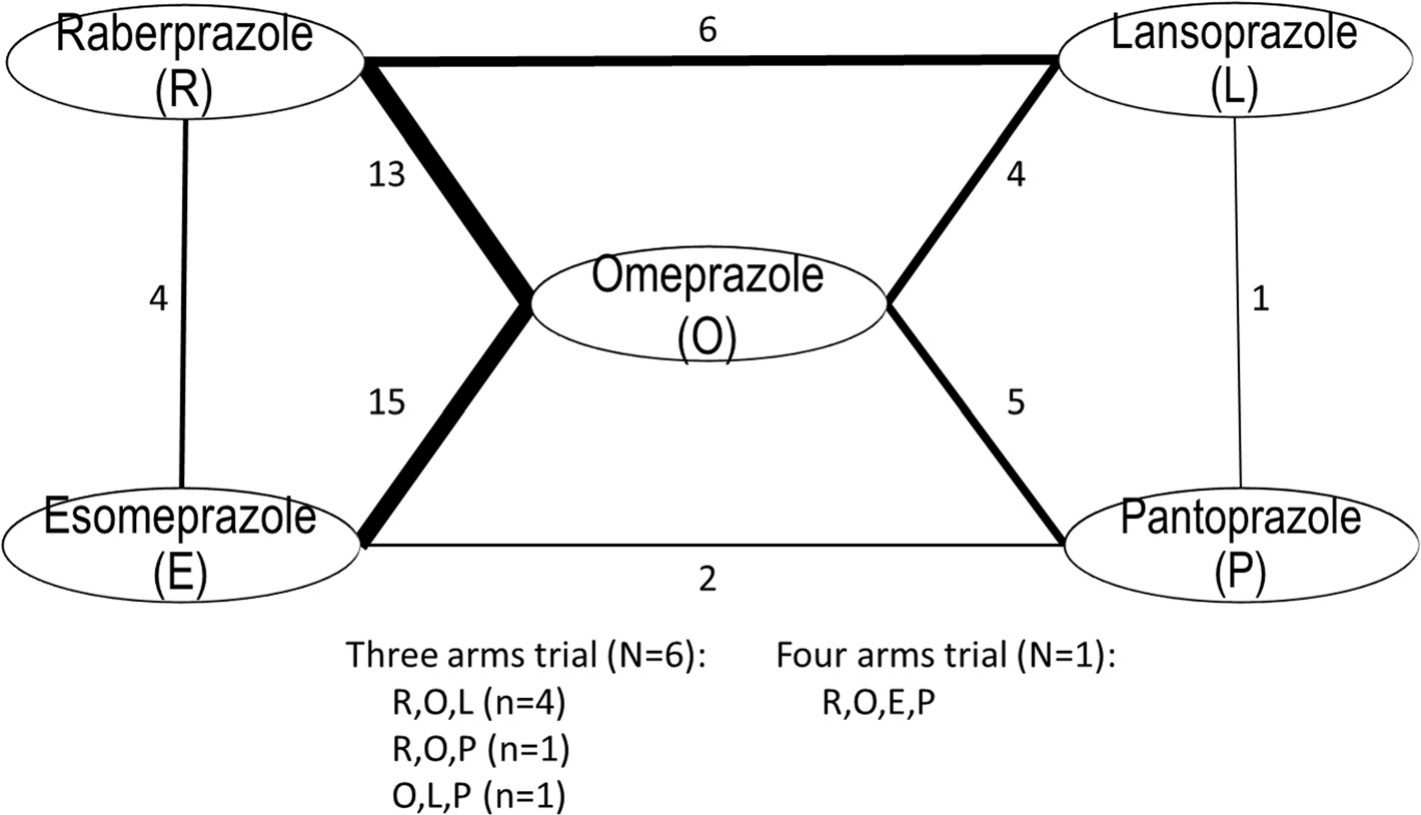
Network diagram. Number represents the number of trials available for that direct comparison

**Fig. 3:**
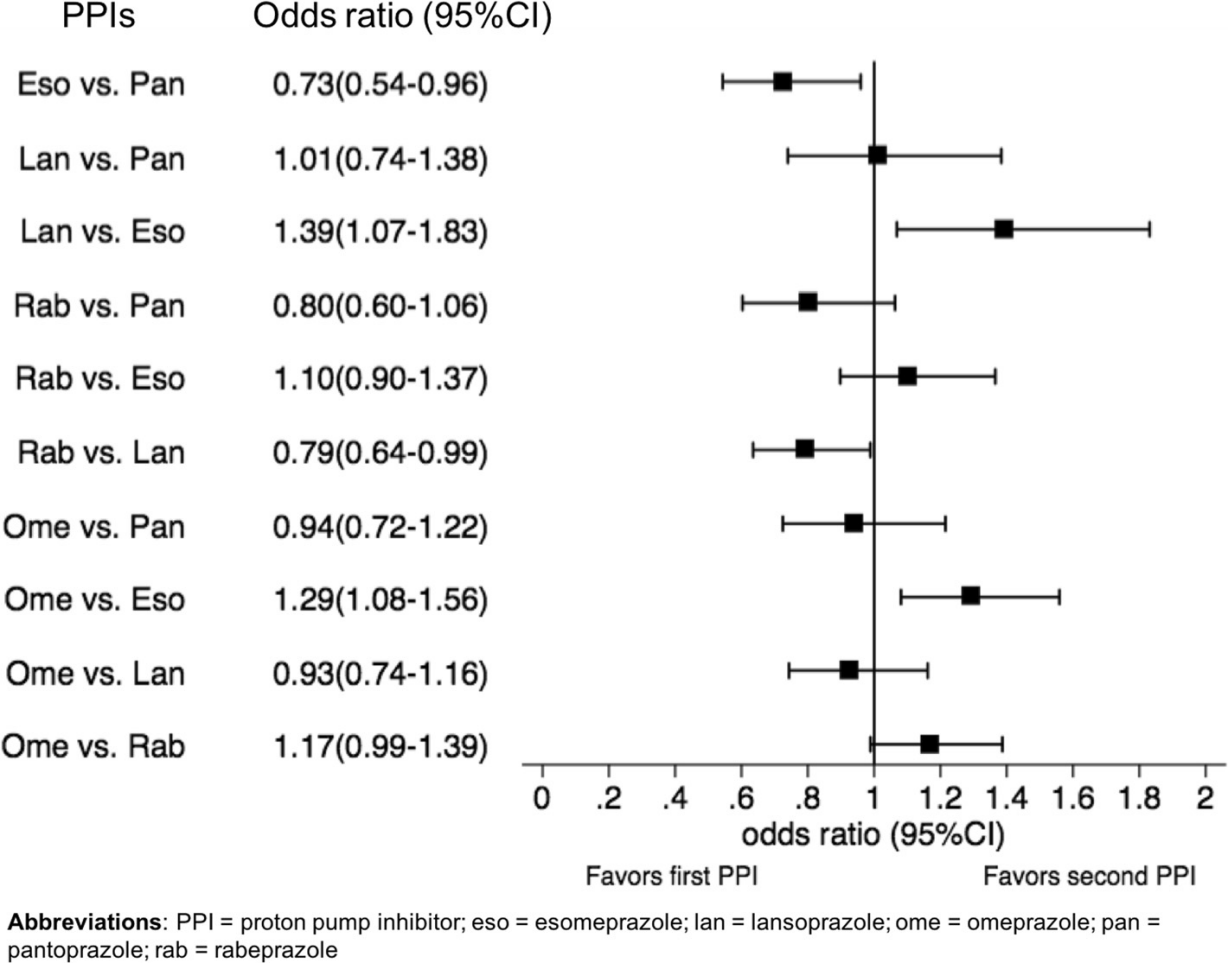
Odds ratios and interval plot of mixed treatment comparisons between PPIs for H.pylori eradication

A diagram of the PPI network is given in Fig. 2. Overall, 57 trials were included in the NMA analysis. None of the trials compared rabeprazole with pantoprazole, or lansoprazole with esomeprazole. In contrast, esomeprazole was compared with omeprazole in 15 trials. In our analysis omeprazole was used as the reference treatment since direct trials existed comparing omeprazole and each of the other PPIs and it was the most commonly used PPI in the triple therapy for H.pylori eradication. Esomeprazole was ranked first in the probability best test, with OR to be 1.29 (95 % credible interval 1.08–1.56) when compared with omeprazole, followed by rabeprazole (Table 2). The three old generations of PPIs showed similar effectiveness. The OR and interval plot for each pair of the mixed comparisons of different PPIs is shown in Fig. 3.

**Table 2:** Rank order of effectiveness of PPIs for H.pylori eradication

Rank	Generation of PPI	PPI	Probability best (standard deviation)	OR (95 % credible Interval) Comparator: Omeprazole
1	New	Esomeprazole	0.820 (0.384)	1.29 (1.08 – 1.56)
2	New	Rabeprazole	0.170 (0.375)	1.77 (0.99 – 1.39)
3	Old	Pantoprazole	0.008 (0.087)	0.94 (0.72 – 1.22)
4	Old	Lansoprazole	0.003 (0.050)	0.93 (0.74 – 1.16)
5	Old	Omeprazole	0.0003 (0.018)	1

### Triple therapy with different antibiotics

Seven studies evaluated the impact of different antibiotics within a triple therapy for the first-line treatment [25, 26, 33–37] and one study evaluated the antibiotics for both first-line and second-line treatment [47] (Table 3). Clarithromycin was used as a comparator in all the studies while the intervention antibiotics included levofloxacin [25, 26, 35–37], azithromycin [33] and moxifloxacin [34]. Five studies compared levofloxacin-based triple therapy with standard triple therapy for first-line treatment [25, 26, 35–37], among which two studies reported improved eradication rates with levofloxacin [25, 26] while the other three studies showed no difference between the two regimens [35–37]. Similarly, for moxifloxacin, two systematic reviews reached conflict conclusions when comparing it with standard triple therapy for first-line treatment [34, 47]. The two systematic reviews included three same RCTs while one of them included an additional RCT from China [34]. With the inclusion of this RCT, the pooled result showed moxifloxacin was associated with greater eradication rate for the naïve patient (OR 1.13; 95%CI 1.01–1.27) [34] while no difference was shown in another study (OR 1.80; 95%CI 0.71–4.55) [47]. The use of moxifloxacin as second-line treatment was evaluated in one study which showed that the moxifloxacin-based triple therapy achieved greater eradication rate than the clarithromycin-based therapy (OR 1.78; 95%CI 1.16–2.73) [47]. In addition to levofloxacin and moxifloxacin, one study evaluated azithromycin-based triple therapy versus standard triple therapy as first-line treatment and did not find a difference [33].

**Table 3:** Characteristics of systematic reviews comparing triple therapy with different antibiotics (*n* = 8)

Author, Year, country	Last search date	Disease	Countries of included RCTs^c^	Intervention	Comparator	No. of studies in MA	No. of patients in MA	Eradication rates by ITT	Eradication rates odds ratio (95 % CI) by ITT	Quality assessment^b^
Zhang et al. 2008 China [25]	May 2008	HP infection; naïve to treatment; PUD/NUD	China (8), Italy (3)	**Levofloxacin-containing triple**: levofloxacin+	**Standard triple**: clarithromycin+	11	1926	Not reported	1.56 (1.25–1.94)	0
				+same PPI(Ome/panto/esome) + another one antibiotic (furazolidone/amoxicillin/azithromycin/metronidazole/tinidazole)						
Dong et al. 2009 China [33]	May 2009	HP infection; naïve to treatment	China (4), Italy (5), Korea, Russia, France, Croatia, US	**Azithromycin-containing triple**: azithromycin+ + one antibiotic (levofloxacin/amoxicillin/metronidazole)+	**Azithromycin NOT-containing triple**: + two antibiotics (amoxicillin/clarithromycin/metronidazole/tinidazole)+	14	1431	72.0 % vs. 69.8 %	1.17 (0.64–2.14)	+
				+same PPI (ome/esome/lanso/panto)						
Yuan et al. 2009 China [34]	Dec 2008	HP infection; naïve to treatment	Italy, Croatia, Turkey, China	**Moxifloxacin-containing** triple: moxifloxacin+	**Clarithromycin-containing** triple: clarithromycin+	4	772	84.1 % vs. 73.6 %	1.13 (1.01–1.27)^a^	+
				+ same PPI (esome/lanso/ome) + another same regimen (amoxicillin/tinidazole/metronidazole/bismuth-)						
Zhang et al. 2013 China [47]	March 2012	HP infection; PUD/NUD/others; either naive or with previous treatment failures	Korea (2), Croatia (2), China, Italy, Turkey	**Moxifloxacin-containing triple OR Quadruple**: moxifloxacin + amoxicillin/metronidazole/tinidazole +/− RBC+	**Standard triple or quadruple**: (+/−)Bismuth/RBC + metronidazole/tinidazole/clarithromycin/amoxicillin+	7	1263	79.0 % vs. 68.3 %	1.82 (1.17–2.81)	+
				+ same PPI (esome/ome/rabe/lanso)						
			Croatia, Turkey, Italy	**First-line**	**First-line**	3	717	Not reported	1.80 (0.71–4.55)	
				**Moxifloxacin-containing triple OR Quadruple**: moxifloxacin + amoxicillin/metronidazole/tinidazole (+/−) RBC+	**Standard triple or quadruple**: (+/−) Bismuth/RBC + metronidazole/tinidazole/clarithromycin/amoxicillin+					
				+ same PPI (esome/lanso)						
			Croatia, Korea (2), China	**Second-line**	**Second-line**	4	546	73.3 % vs. 60.2 %	1.78 (1.16–2.73)	
				**Moxifloxacin-containing triple:** moxifloxacin + metronidazole/amoxicillin+	**Standard triple or quadruple**: (+/−) Bismuth + metronidazole/tinidazole/clarithromycin+					
				+ same PPI (esome/ome/rabe)						
			Croatia, Korea (2), Turkey, Italy, China	**Moxifloxacin + amoxicillin** (+/−) RBC+	**Standard triple or quadruple**: (+/−) Bismuth/RBC + metronidazole/tinidazole/clarithromycin/amoxicillin+	6	810	Not reported	1.50 (0.95–2.38)	
				+ same PPI(esome/ome/rabe/lanso)						
			Croatia (2), Italy	**Moxifloxacin + metronidazole/tinidazole+**	**Standard triple or quadruple**: (+/−) Bismuth/RBC + metronidazole/tinidazole/clarithromycin/amoxicillin+	3	453	Not reported	3.00 (1.84–4.89)	
				+ same PPI (esome/ome/rabe/lanso)						
Ye et al. 2014 China [35]	August 2013	HP infection; naïve to treatment	Germany, Egypt, Taiwan (2), China (2), Spain (2), Italy (2)	**Levofloxacin-containing triple**: levofloxacin+	**Standard triple**: clarithromycin+	10	2676	81.5 % vs. 77.2 %	1.28 (0.88–1.85)	++
				+same PPI(Ome/lanso/esome) + another one antibiotic (amoxicillin/metronidazole)						
Peedikayil et al. 2014 Saudi Arabia [36]	March 2013	HP infection; naïve to treatment	Egypt and Saudi Arabia, Taiwan (2), South Korea, China, Italy (2)	**Levofloxacin-containing triple**: levofloxacin+	**Standard triple**: clarithromycin+	7	1782	79.1 % vs. 81.4 %	0.97 (0.93–1.02)^a^	+
				+same PPI(Ome/lanso/esome) + another one antibiotic (amoxicillin/+metronidazole/clarithromycin/azithromycin)						
Xiao et al. 2014 China [37]	March 2013	HP infection; naïve to treatment, PUD/NUD/not reported	Italy (2), China (3), Spain (2), Egypt and Saudi Arabia, Korea	**Levofloxacin-containing triple**: levofloxacin+	**Standard triple**: clarithromycin+	9	2512	80.2 % vs. 77.4 %	1.03 (0.94–1.13)^a^	++
				+same PPI(Ome/lanso/esome) + another one antibiotic (amoxicillin/+metronidazole/clarithromycin/azithromycin)						
Gou et al. 2014 China [26]	December 2013	HP infection; naïve to treatment	All from China	**Levofloxacin-containing triple**: levofloxacin+	**Standard triple**: clarithromycin+	21	2697	82.3 % vs.73.8 %	1.12 (1.08–1.16)^a^	0
				No details reported						

*HP* H.pylori, *PPI* proton pump inhibitor, *esome* esomeprazole, *lanso* lansoprazole, *ome* omeprazole, *panto* pantoprazole, *rabe* rabeprazole, *PUD* peptic ulcer disease, *NUD* non-ulcer dyspepsia, *MA* meta-analysis, *ITT* intention to treat, *CI* confidence interval, *RCT* randomized controlled trials, *RBC* ranitidine bismuth citrate

^a^ Relative risk is reported here

^b^ Quality assessment: high quality (++): majority of criteria met, little or no risk of bias and results unlikely to be changed by further research. Acceptable (+): most criteria met, some flaws in the study with an associated risk of bias and conclusions may change in the light of further studies. Low quality (0): either most criteria not met or significant flaws relating to key aspects of study design, and conclusions likely to change in the light of further studies

^c^ Countries of included RCTs: the number in the bracket represents the number of trials from the same country if more than one trial exists

In addition to the eradication rates, adverse events rates were also compared in seven studies, such as nausea, metallic taste and other gastrointestinal tract discomforts [25, 33–37, 47]. Compared to clarithromycin, the risk of adverse events was approximately halved with azithromycin (OR 0.58; 95%CI 0.41–0.82) [33]. Two studies compared the adverse events between moxifloxacin and clarithromycin containing triple therapy; one showed lower adverse events rate associated with moxifloxacin (OR 0.45; 95%CI 0.26–0.77) [47] while the other did not show any difference [34]. For levofloxacin, one study showed there were reduced adverse events rate (OR 0.57; 95%CI 0.44–0.74) [25] while three studies reported no difference when comparing to the standard therapy [35–37].

### Triple therapy versus bismuth-based therapy

Nine studies compared the effectiveness, adverse events rate and therapy discontinuation rates between triple therapy and bismuth-based therapy [27–32, 38, 39, 50]. The study characteristics are presented in Table 4. For the bismuth-based therapies, seven studies evaluated bismuth-based quadruple therapy [29–32, 38, 39, 50], one evaluated ranitidine bismuth citrate (RBC) [27] and another one evaluated both quadruple therapy and RBC [28]. Overall, the quadruple therapy was associated with similar or greater eradication rate than standard triple therapy; however when levofloxacin or moxifloxacin was contained in the triple therapy, the reverse was observed.

**Table 4:** Characteristics of systematic reviews comparing triple therapy versus bismuth-based therapy (*n* = 9)

Author, Year, country	Last search date	Disease	Countries of included RCTs^f^	Triple therapy	Bismuth-based Quadruple therapy	No. of studies in MA	No. of patients in MA	Eradication rates by ITT	Eradication rates odds ratio (95 % CI) by ITT^d^	Quality assessment^e^
Gene et al. 2003 Spain [38]	Aug 2002	HP infection; naïve to therapy; PUD/NUD	Spain (2), US/Canada, unknown	PPI (ome/panto) + clarithromycin + amoxicillin	Bismuth + PPI(ome/panto) + tetracycline + metronidazole	4	981	78 % vs. 81 %	0.83 (0.61–1.14)^a^	0
Gisbert et al. 2005 Spain [27]	Sep 2004	HP infection; NUD+/−PUD; previous treatment failures	Croatia, Spain (6), Belgium, Italy (4), Greece, China	PPI (ome/lanso/panto) + clarithromycin + amoxicillin/nitroimidazole	RBC + **clarithromycin + amoxicillin**	14	2205	78 % vs. 79 %	**Bismuth vs. triple** 1.11 (0.88–1.40)	+
			Croatia, Italy (6), Spain, Norway, Unknown (2), The Netherlands, China	PPI (ome/lanso/panto/rabe) + clarithromycin + amoxicillin/nitroimidazole	RBC + **clarithromycin + nitroimidazole**	13	1777	80 % vs. 87 %	**Bismuth vs. triple** 1.65 (1.15–2.37)	
			Taiwan, China, UK	PPI (ome) + clarithromycin + amoxicillin/nitroimidazole	RBC + **nitroimidazole + amoxicillin**	3	451	75 % vs. 73 %	**Bismuth vs. triple** 0.92 (0.60–1.41)	
Gisbert et al. 2006 Spain [28]	Jul 2005	HP infection; Previous treatment failures	Italy (5), China, Spain, unknown	**Levofloxacin-containing**: levofloxacin + PPI(panto/rabe/esome/ome) + amoxicillin/rifabutin	Bismuth + PPI(panto/rabe/ome) + tetracycline + metronidazole; or RBC+ tetracycline + metronidazole	8	996	81 % vs. 70 %	1.80 (0.9–3.5)	0
			Not reported	**Levofloxacin + amoxicillin** + PPI(panto/rabe/esome/ome)	Bismuth + PPI(panto/rabe/ome) + tetracycline + metronidazole; or RBC+ tetracycline + metronidazole	not specified	Not specified	Not reported	1.7 (0.71–4.0)	
Saad et al. 2006 US [29]	Apr 2005	HP infection; failed prior course(s) of standard triple therapy	Italy (5), China	**Levofloxacin-containing** : levofloxacin+ +amoxicillin+	Bismuth − + metronidazole + tetracycline+	6	854	87 % vs. 60 %	1.18 (1.08–1.29)^b^	0
				+ same PPI (ome/esome/rabe/panto)						
Li et al. 2010 China [30]	1981-Mar 2009 (Published date)	HP infection; previous treatment failures	Germany (2), Ireland	**Clarithromycin-containing**: clarithromycin + amoxicillin+	Bismuth+ +metronidazole + tetracycline+	3	411	46.5 % vs. 61.9 %	0.53 (0.35–0.80)	0
				+ same PPI (ome/not specified)						
			Korea (2), Croatia	**Moxifloxacin-containing:** moxifloxacin + amoxicillin/metronidazole+	Bismuth + metronidazole + tetracycline+	3	437	Not reported	1.78 (0.98–3.22)	
				+PPI(esome/ome)						
			Taiwan, Korea, China (5), Italy (2)	**Levofloxacin-containing**: levofloxacin + amoxicillin/rifabutin+	Bismuth + metronidazole + tetracycline+	9	928	Not reported	1.43 (0.82–2.51)	
				+ same PPI(esome/panto/lanso/rabe)						
Luther et al. 2010 US [50]	1990–2008 (Published date)	HP infection	Spain (2), Greece, Australia/New Zealand, India, US/Canada, Korea, Turkey, UK	**Clarithromycin-containing:** clarithromycin + amoxicillin+	Bismuth + metronidazole + tetracycline +	9	1679	77.0 % vs. 78.3 %	**Bismuth vs. triple** 1.00 (0.94–1.07^b^	0
				+PPI (ome/panto/lanso/not specified)						
Wu et al. 2011 China [31]	Dec 2010	HP infection; previous treatment failures	China (4), Korea (2), Croatia	**Moxifloxacin-containing**: Moxifloxacin + + amoxicillin/metronidazole+	Bismuth + metronidazole/furazolidone + tetracycline/amoxicillin/clarithromycin+	7	787	74.9 % vs. 61.4 %	1.89 (1.38–2.58)	++
				+ PPI (esome/ome/rabe)						
Di Caro et al. 2012 UK [32]	Oct 2010	HP infection; previous treatment failures	Italy (4), Spain (2), China (4), Korea (2), Taiwan, Unknown	**Levofloxacin + amoxicillin-containing**: levofloxacin + amoxicillin + PPI(panto/rabe/esome/ome/lanso)	Bismuth quadruple therapy (not specified)	14	1331	76.5 % vs. 67.4 %	1.59 (0.98–2.58)	0
Venerito et al. 2013 Germany [39]	Nov 2011	HP infection; naïve to therapy; PUD/NUD/others	Spain (2), Australia/New Zealand, Greece, US/Canada, India, Korea, Turkey (2), UK, China, multi European countries	**Clarithromycin-containing:** clarithromycin + amoxicillin+	Bismuth + tetracycline + metronidazole+	12	2467	68.9 % vs. 77.6 %	**Bismuth vs. triple** 0.06 (−0.01–0.13)^c^	+
				+PPI(ome/panto/lanso/not specified)						

*HP* H.pylori, *PPI* proton pump inhibitor, *esome* esomeprazole, *lanso* lansoprazole, *ome* omeprazole, *panto* pantoprazole, *rabe* rabeprazole, *PUD* peptic ulcer disease, *NUD* non-ulcer dyspepsia, *MA* meta-analysis, *ITT* intention to treat, *CI* confidence interval, *RCT* randomized controlled trials, *RBC* ranitidine bismuth citrate

^a^ Peto OR is reported here

^b^ Relative risk is reported here

^c^ Risk difference is reported here

^d^ OR > 1 indicates that triple therapy is associated with greater effectiveness than bismuth-based therapy and vice versa. When “Bismuth vs. triple” is specified in the form, OR > 1 indicates bismuth-based therapy is associated with greater effectiveness than triple therapy and vice versa

^e^ Quality assessment: high quality (++): majority of criteria met, little or no risk of bias and results unlikely to be changed by further research. Acceptable (+): most criteria met, some flaws in the study with an associated risk of bias and conclusions may change in the light of further studies. Low quality (0): either most criteria not met or significant flaws relating to key aspects of study design, and conclusions likely to change in the light of further studies

^f^ Countries of included RCTs: the number in the bracket represents the number of trials from the same country if more than one trials exist

Two of the nine studies focused on treatment naive patients, and no difference in eradication rates was found between triple and quadruple therapy. The primary antibiotics used in both studies was clarithromycin [38, 39]. The remaining seven studies compared second-line therapy for patients with previous treatment failures [27–32, 50]. The primary antibiotics used in triple therapy varied: two studies evaluated clarithromycin [27, 50], one study with moxifloxacin [31], three studies with levofloxacin [28, 29, 32], and one study compared all of the three [30]. Clarithromycin-containing triple therapy was associated with lower eradication rates than bismuth-based therapy in two studies [27, 30] while one study showed no difference [50]. In contrast, moxifloxacin-containing triple therapy was suggested to achieve greater effectiveness than bismuth-based therapy [30, 31]. Similar to moxifloxacin, triple therapy with levofloxacin appeared to be more effective than bismuth-based therapy, however statistically significant finding was only reported in one of the four studies (OR 1.18; 95%CI 1.08–1.29) [29].

The adverse events around the bismuth-based therapy included diarrhea, abdominal pain, dark stools, dizziness, headache, nausea, metallic taste and nausea [52]. Seven studies reported adverse events of the compared regimens [28–32, 38, 50]. The pooled adverse event rates of clarithromycin-based triple therapy ranged from 35.4 % [50] to 37 % [38], 10.1 % [31] to 16.75 % [30] for levofloxacin or moxifloxacin-based triple therapy, and 27.8 % [31] to 44 % [28] for bismuth-based therapy. Five studies showed a lower risk of adverse events of levofloxacin/moxifloxacin compared with the bismuth-based regimen with ORs ranging from 0.27 to 0.51 [28–32], and one study favoured bismuth when comparing with clarithromycin triple therapy [50]. One study classified adverse events by severity and reported much lower risk favouring levofloxacin compared with bismuth therapy when including only severe adverse events (OR 0.20; 95%CI 0.06–0.67) [28]. Furthermore, the discontinuation rate of triple therapy using moxifloxacin and levofloxacin was statistically significantly lower than bismuth-based therapy in three of the four studies [29–31].

### PPI versus H_2_RA in triple therapy

Three studies compared the effectiveness of PPI versus H_2_RA within a triple therapy (Table 5) [40, 41, 53]. One systematic review based on 20 RCTs with 2374 patients showed PPI was associated with greater effectiveness than H_2_RA (OR 1.31; 95%CI 1.09–1.58) [40]. Another study of 12 RCTs did not show any difference between the two, but its subgroup analysis based on six RCTs suggested PPI-based triple therapy reached higher eradication rates than H_2_RA when clarithromycin was not contained [53]. A recent systematic review of three RCTs compared lafutidine versus lansoprazole-containing triple therapy and reported no difference between the two regimens [41].

**Table 5:** Characteristics of systematic reviews comparing PPI and H_2_ receptor antagonists (H_2_RAs) (*n* = 3)

Author, year, country	Last search date	Disease	H_2_RAs	PPI	No. of studies in MA	No. of patients in MA	Eradication rates by ITT	Eradication rates odds ratio (95 % CI) by ITT	Quality assessment^a^
Gisbert et al. 2003 Spain [40]	Jan 2002	HP infection; naïve to treatment; PUD/NUD	H_2_RAs (ranitidine/famotidine/nizatidine)+	PPI (ome/lanso)+	20	2374	69 % vs. 74 %	**Triple vs. H**_**2**_ **RAs** 1.31 (1.09-1.58)	+
			+ two same antibiotics (amoxicillin/clarithromycin/metronidazole/tinidazole) +/− bismuth-						
Graham et al. 2003 US [53]	1990–2001 (Published date)	HP infection; either naïve or with previous treatment failures	H_2_RAs(nizatidine/famotidine/ranitidine) +	PPI (lanso/ome) +	12	1441	78 % vs. 81 %	0.83 (0.63–1.09)	0
			+ two same antibiotics (clarithromycin/amoxicillin/metronidazole/tinidazole)						
			H_2_RAs(not specified)+	**Clarithromycin-containing triple** : Clarithromycin + PPI(not specified)+	6	Not reported	79 % vs. 69 %	1.14 (0.76–1.71)	
			+ one same antibiotics (not specified)						
			H_2_RAs(not specified)+	**Clarithromycin NOT-containing triple**: PPI(not specified)+	6	Not reported	78 % vs. 85 %	0.64 (0.45–0.92)	
			+two same antibiotics (not specified)						
Ren et al. 2010 China [41]	Apr 2010	HP infection; naïve to treatment	**Lafutidine-containing:** H_2_RAs(lafutidine)+	**Lanso-containing triple**: PPI(lanso) +	3	238	78 % vs. 77.5 %	1.03 (0.64–1.66)	++
			+ two same antibiotics (clarithromycin + amoxicillin)						

*HP* H.pylori, *H2RAs* H2 receptor antagonists, *PPI* proton pump inhibitor, *esome* esomeprazole, *lanso* lansoprazole, *ome* omeprazole, *panto* pantoprazole, *rabe* rabeprazole, *PUD* peptic ulcer disease, *NUD* non-ulcer dyspepsia, *MA* meta-analysis, *ITT* intention to treat, *CI* confidence interval, *RCT* randomized controlled trials

^a^ Quality assessment: high quality (++): majority of criteria met, little or no risk of bias and results unlikely to be changed by further research. Acceptable (+): most criteria met, some flaws in the study with an associated risk of bias and conclusions may change in the light of further studies. Low quality (0): either most criteria not met or significant flaws relating to key aspects of study design, and conclusions likely to change in the light of further studies

### Other drug therapies

One study evaluated the impact of adding metronidazole or tinidazole (concomitant quadruple therapy) on standard triple therapy and reported greater eradication rates with concomitant therapy (OR 2.36; 95%CI 1.67–3.34) [48]. One study based on 14 RCTs assessed the combination of tetracycline and amoxicillin in triple therapy/quadruple therapy and found no difference in eradication rate when compared to other regimens when the two drugs were not combined [49]. One Japanese study evaluated the effectiveness of supplementation with rebamipide and found it was associated with greater eradication rate compared to rebamipide not-containing regimens (OR 1.59; 95%CI 1.14–2.22) [54]. The characteristics of these studies are presented in Table 6.

**Table 6:** Characteristics of systematic reviews comparing other regimens (*n* = 3)

Author, year, country	Last search date	Disease	Countries of included RCTs^b^	Intervention	Comparison	No. of studies in MA	No. of patients in MA	Eradication rates by ITT	Eradication rates odds ratio (95 % CI) by ITT	Quality assessment^a^
Gisbert and Calvet 2012 Spain [48]	December 2011	HP infection PUD/NUD/others	Germany, UK, Japan, Italy, Japan, Korea (2)	Concomitant therapy: **metronidazole** + standard triple therapy	Standard triple therapy	7	984	90 % vs. 78 %	2.36 (1.67–3.34)	0
				Note: Standard triple therapy: (PPI(ome/rabe/lanso) + amoxicillin + clarithromycin)						
Lv et al. 2015 China [49]	April 2014	HP infection; PUD/NUD/others; naïve to treatment or had previous treatment	China (4), Taiwan (3), Korea, Turkey	**Quadruple** regimens containing **both amoxicillin and tetracycline**	Other quadruple regimens where amoxicillin and tetracycline were not contained together	9	1453	78.1 % vs. 80.5 %	0.90 (0.46–1.78)	+
			US, Italy, Turkey, Taiwan, China	**Triple** therapy containing both **amoxicillin and tetracycline**	Other regimens where amoxicillin and tetracycline were not contained together	5	840	68.8 % vs. 66.7 %	1.21 (0.64–2.28)	
Nishizawa et al. 2014 Japan [54]	July 2014	HP infection	Japan (5), Korea	**Rebamipide containing regimen:** rebamipide+	**Rebamipide NOT-containing regimen**: none or mucosal protective agents other than rebamipide (teprenone/plaunotol)+	6	611	63.5 % vs. 52.7 %	1.59 (1.14–2.22)	+
				+PPI(lanso/ome) + antibiotics (amoxicillin/metronidazole)						

*HP* H.pylori, *PPI* proton pump inhibitor, *esome* esomeprazole, *lanso* lansoprazole, *ome* omeprazole, *panto* pantoprazole, *rabe* rabeprazole, *PUD* peptic ulcer disease, *NUD* non-ulcer dyspepsia, *MA* meta-analysis, *ITT* intention to treat, *CI* confidence interval, *RCT* randomized controlled trials

^a^ Quality assessment: high quality (++): majority of criteria met, little or no risk of bias and results unlikely to be changed by further research. Acceptable (+): most criteria met, some flaws in the study with an associated risk of bias and conclusions may change in the light of further studies. Low quality (0): either most criteria not met or significant flaws relating to key aspects of study design, and conclusions likely to change in the light of further studies

^b^ Countries of included RCTs: the number in the bracket represents the number of trials from the same country if more than one trials exist

### Quality assessment

**Fig. 4:**
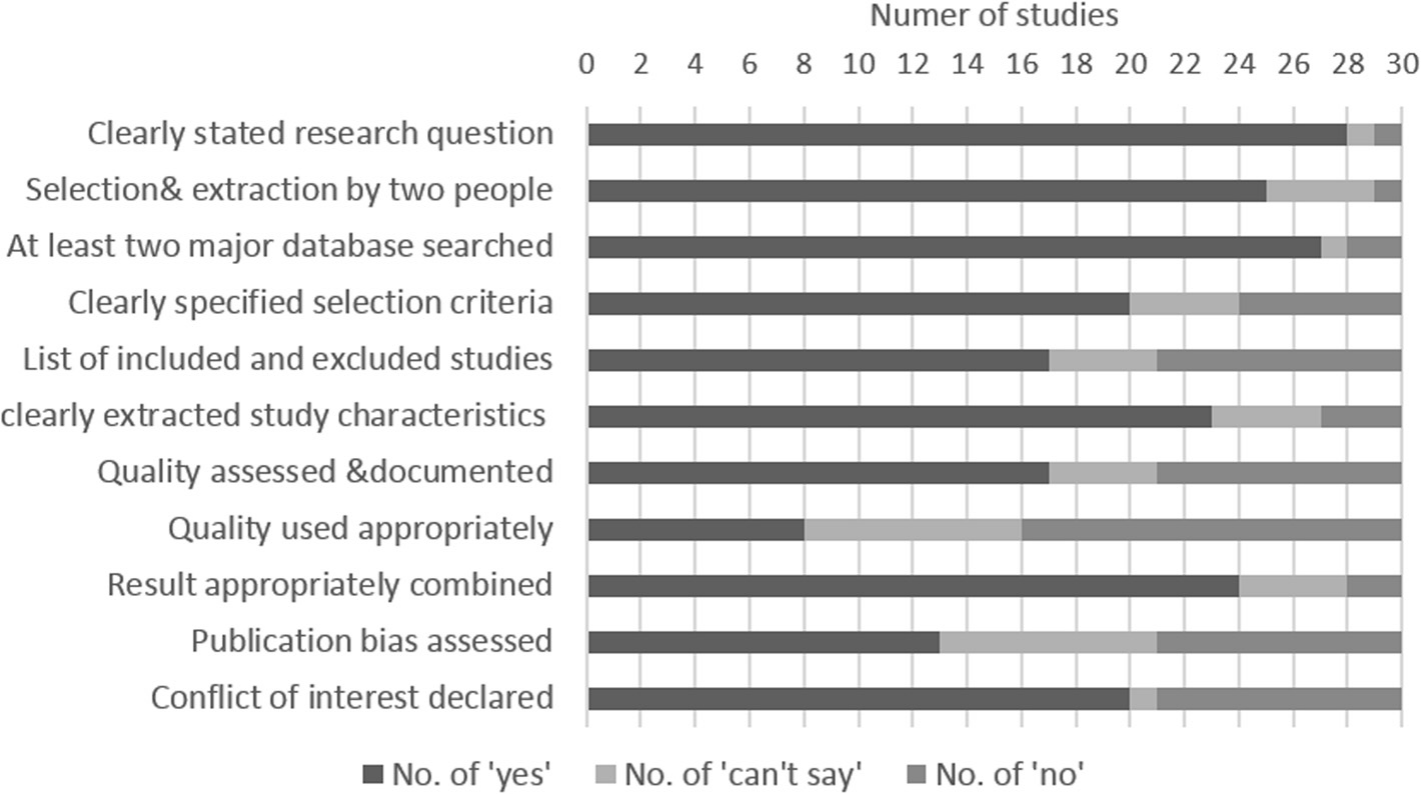
Overall performance of included systematic reviews for each AMSTAR critical appraisal criteria

The overall quality of the included systematic reviews was graded as low to moderate with a higher risk of bias (Fig. 4). This was primarily due to insufficient reporting and poor methodological approaches. The majority of the reviews met five to eight criteria out of the 11 total AMSTAR criteria. The criteria that were frequently not fulfilled included: (1) transparent study selection process and reference of excluded studies; (2) adequate reporting of the population characteristics; (3) using quality appropriately in making conclusions; (4) assessing publication bias when applicable. The detailed assessment for each of the included studies is presented in Additional file 4: Table S2.

## Discussion

### Summary of findings

This overview of systematic reviews evaluated the effectiveness of pharmacological regimens for the eradication of H.pylori by searching and analysing the existing systematic reviews from 2002 to present. In triple therapy, regarding the use of different PPIs, we found that the results of studies were inconsistent; however more recently published studies tend to suggest new generation PPIs were associated with greater eradication rates than the old generation. The NMA suggested that esomeprazole was the most effective PPI with the highest probability to be the best among the five PPIs after incorporating evidence of both direct and indirect comparisons. Regarding the use of antibiotics, conflicting results exist between the studies to some extent; however this could be due to the varied resistant rate to different antibiotics across regions. This leads to the limited transferability of RCT results across countries and population and thus, there exist issues of fundamental heterogeneity when pooling results together in the meta-analysis. Concerning the comparison between triple therapy and bismuth-based therapy, there was no difference between the two regimens overall, but the antibiotics within the triple therapy may have an impact on the overall effectiveness of the drug regimen. Moxifloxacin or levofloxacin based triple therapy were associated with greater eradication rates, lower risk of adverse events and lower discontinuation rate than bismuth-based therapy for second-line treatment. With regard to the comparison between triple therapies and H_2_ receptor antagonist and others, no definite conclusion could be reached due to limited available evidence.

The evidence on the effectiveness of PPI has evolved over time. Contrary to existing guidance, recent studies have shown that the new generation PPIs have achieved statistically significant greater effectiveness rate than the old generations. There is a clear time trend when evaluating the systematic reviews – systematic reviews published before 2006 reported no difference, while 2006 onwards, the statistical significant difference was shown in the pooled results. This can be explained by more recent RCTs and a more complete evidence base included in the recent systematic reviews (Additional file 5: Table S3). When comparing between triple therapy and bismuth-based therapy, the results were mixed. However, there seems to be a trend according to the choice of antibiotics in triple therapy – triple therapy achieved greater eradication rates than bismuth-based therapy when moxifloxacin or levofloxacin was used as a substitute of clarithromycin for second-line treatment. Although generally the results of comparing triple therapy and bismuth-based therapy failed to show statistical significance, it is possible this is a sample size issue. Our results support the current guidance on the recommendations of moxifloxacin or levofloxacin as the second-line treatment for previous treatment failures of H.pylori. However, its role as a first-line therapy was found to be controversial. Two studies showed the use of levofloxacin or moxifloxacin for treating naïve patients was associated with improved eradication rate [25, 26], while three studies found no difference [35–37]. This was further investigated by two subgroup analyses from two included studies which both suggested that levofloxacin achieved statistically greater eradication rates in European countries where the resistant rates were much lower than the global average [35, 37]. Therefore, the discrepancy of the results could be attributed to the varied resistant rates to different antibiotics across regions or populations. This could also possibly explain that the two meta-analyses which pooled RCTs mostly from China showed the improved effectiveness of levofloxacin as first-line treatment [25, 26] – the resistant rate to clarithromycin could be possibly much higher than that to levofloxacin in the regions where the included RCTs were conducted.

### Comparison with current guidelines

Based on current guidelines from the American College of Gastroenterology, Canadian Helicobacter Study Group and National Institute for Health and Care Excellence (NICE), a triple regimen consisting of a PPI, clarithromycin with either metronidazole or amoxicillin is recommended as first-line treatment [13–15]. In addition, both of the American and Canadian guidelines recommend the combination of PPI, bismuth, tetracycline and metronidazole as an alternative for first-line therapy [13, 14]. The alternative of bismuth quadruple therapy is raised due to the increasing clarithromycin resistance rate which has lowered the efficacy of triple therapy to 70–85 %. The American guideline also recommends to consider levofloxacin-based triple therapy when bismuth or clarithromycin-based therapies are not an option in some circumstances [14]. In 2009, the Asia–Pacific H.pylori Consensus Conference agreed that the first-line treatment should consist of either clarithromycin-based triple or bismuth quadruple therapy, and further proposed four options for second-line treatment: (i) standard triple therapy that has not been previously used; (ii) bismuth-based quadruple therapy; (iii) levofloxacin-based triple therapy; and (iv) rifabutin-based triple therapy [55].

The European Helicobacter Study Group published their latest guideline in 2012 – the Maastricht IV report [12] recommending specific H.pylori eradication strategies according to different clarithromycin resistance rates. The threshold for classifying clarithromycin resistance to the high/low area is set as 15 % –20 %. In regions with low clarithromycin resistance rates, clarithromycin-based triple therapy remains the first-line treatment, with the alternative of bismuth quadruple therapy. Where there is higher clarithromycin resistance, the bismuth-based therapy is recommended as the first-line treatment. In both circumstances, levofloxacin is recommended for second-line therapy rather than first-line for the reason of ’rapid acquisition of resistance’.

The World Gastroenterology Organization published their H.pylori guideline for developing countries in 2011, which is consistent with the above guidelines. However, it states that, due to its low cost, furazolidone may be served as an alternative option by developing countries, such as Brazil and China, despite being withdrawn in the US and the European Union due to the severe adverse events [56].

It is worth noting that the type of PPI is not specified in any of the current guidelines, which may be due to the limited availability of reliable evidence from studies when those guidelines were published. However, our review showed that the esomeprazole could achieve greater eradication rate than the older generation of PPIs. Despite the relatively high cost of newer generation of PPI, this difference in effectiveness between the generations of PPIs should be taken into account in the recommendations. Our finding supported the recommendation of bismuth-based therapy as a first-line alternative to standard triple therapy in a high clarithromycin resistant area. For the second-line treatment, our findings are consistent with the current guidelines; both moxifloxacin/levofloxacin containing triple therapy and bismuth-based therapy can achieve higher eradication rates than clarithromycin-containing triple therapy. Moreover, the former appeared to be superior to the latter in terms of eradication rates and adverse events rates.

### Limitations

There are a few limitations of this study. As this is an overview of systematic reviews, our results are dependent on what has been reported in the included systematic reviews and on the methodological rigour applied in their development. For instance, similar search strategy across systematic reviews has turned out to include difference RCTs. The low-moderate overall quality of included studies may affect the impact of this overview of systematic reviews on clinical decision making. However, it is difficult to judge whether the low internal validity of the individual systematic reviews resulted from insufficient reporting or certain methodological flaws. In addition, there were some heterogeneity issues in this overview. The systematic reviews have included a mixture of population characteristics, countries of origin and comorbidities, infection epidemiology and antibiotics resistance type and thus the eradication rates varied with those factors. This may not be appropriately considered and addressed in some of the included meta-analysis, leading to the inconsistent results in our findings.

## Conclusions

This overview of systematic reviews suggests that the new generation of PPIs and use of moxifloxacin or levofloxacin in triple therapy or bismuth-based therapy as second-line treatment were associated with greater effectiveness, while the comparative effectiveness of antibiotics is complex which probably depends on the resistant rate to different antibiotics in different regions. This should be explored in future research for updating the guidelines. In addition, considering the substantiated difference in the cost of treatment, estimating the cost-effectiveness of these treatments is of value to clinical decision making, especially in the area with high H.pylori prevalence. Given the variation in infection epidemiology and increasing antibiotics resistance, from a clinical perspective, the recommendations should be localized based on the specific prevalence of H.pylori infection and antibiotics resistance rate in the local region and population.

### Abbreviations

AMSTAR, A measurement tool to assess systematic reviews; CI, Confidence interval; CrI, Credible interval; H.pylori, Helicobacter pylori; H_2_RA, H_2_ receptor antagonist; ITT, Intention to treat; NICE, National Institute for Health and Care Excellence; NMA, Network meta-analysis; OR, Odds ratio; PPI, Proton pump inhibitor; RBC, Ranitidine bismuth citrate; RCT, Randomized controlled trial; SIGN, Scottish Intercollegiate Guidelines Network; UK, United Kingdom; US, United States

## Additional files

Additional file 1: Search strategy. (DOCX 13 kb) Additional file 2: WinBUGS code for the network meta-analysis model. (DOCX 12 kb) Additional file 3: Table S1. Excluded studies based on full-text review. (DOCX 14 kb) Additional file 4: Table S2. Quality assessment of included studies based on revised AMSTAR checklist. (DOCX 17 kb) Additional file 5: Table S3. Individual study check table. (DOCX 52 kb)
