# Supplementary material for: Pharmacological regimens for eradication of Helicobacter pylori: an overview of systematic reviews and network meta-analysis
Source: BMC Gastroenterol. 2016 Jul 26;16:80. doi: 10.1186/s12876-016-0491-7 (PMC4962503; doi:10.1186/s12876-016-0491-7)
Supplement: Additional file 1: — Search strategy. (DOCX 13 kb) [file 12876_2016_491_MOESM1_ESM.docx]

**Additional file 1 – Search strategy**

MEDLINE

1. Helicobacter Infections/

2. Helicobacter pylori/

3. h pylori$.mp.

4. h?pylori$.mp.

5. Helicobacter pylori$.mp.

6. campylobacter pylori$.tw.

7. or/1-6

EMBASE

1. Helicobacter Infection/

2. Helicobacter pylori/

3. h pylori$.mp.

4. h?pylori$.mp.

5. Helicobacter pylori$.mp.

6. campylobacter pylori$.tw.

7. or/1-6

Cochrane library

#1 MeSH descriptor: [Helicobacter pylori] explode all trees

#2 MeSH descriptor: [Helicobacter Infections] explode all trees

#3 helicobacter pylori*

#4 h pylori*

#5 h*pylori*

#6 campylobacter pylori*

#7 ([1-#6](#_ENREF_1))
