# Supplementary material for: Pharmacological regimens for eradication of Helicobacter pylori: an overview of systematic reviews and network meta-analysis
Source: BMC Gastroenterol. 2016 Jul 26;16:80. doi: 10.1186/s12876-016-0491-7 (PMC4962503; doi:10.1186/s12876-016-0491-7)
Supplement: Additional file 2: — WinBUGS code for the network meta-analysis model. (DOCX 12 kb) [file 12876_2016_491_MOESM2_ESM.docx]

**Additional file 2 --- WinBUGS code for the network meta-analysis model**

Model code

{

for (i in 1:ns){

w[i, 1] <- 0

delta[i, t[i,1]] <- 0

mu[i] ~ dnorm(0, 0.0001)

for (j in nf[i]:na[i]) {

r[i, j] ~ dbin(p[i,j],n[i, j])

logit(p[i,j])<-mu[i] + delta[i,t[i,j]]

}

# Delta prior, mixed effect comparison and multi-arm adjustment

for (j in 2:na[i]) {

# trial-specific LOR distributions

delta[i, t[i,j]] ~ dnorm(md[i, t[i,j]],precd[i, t[i,j]])

# mean of LOR distributions

md[i, t[i,j]] <- d[t[i,j]] - d[t[i,1]] + sw[i,j]

#precision of LOR distributions

precd[i, t[i,j]] <- prec*2*(j-1)/j

#adjustment, multi-arm RCTs

w[i, j] <- (delta[i, t[i,j]] - d[t[i,j]] + d[t[i,1]])

# cumulative adjustment for multi-arm trials

sw[i, j] <-sum(w[i,1:j-1])/(j-1)

}

}

#Ranking to find best intervention

for (j in 1:nt){

pbest[j]<- equals(rank(d[],j),5)

}

# vague priors for basic parameters

d[1]<-0

for (k in 2:nt){

d[k] ~ dnorm(0,0.0001)

}

#vague priors for random effects standard deviation

#1. Inverse-Gamma

# prec ~ dgamma(0.01, 0.01)

# tau <- 1/sqrt(prec)

# 2. Uniform

tau~dunif(0,10)

prec<-1/(tau*tau)

# pairwise log odds ratios

for (c in 1:(nt-1)){

for (k in (c+1):nt){

lor[c,k] <- d[k] - d[c]

}

}

}
