# Supplementary material for: Pharmacological regimens for eradication of Helicobacter pylori: an overview of systematic reviews and network meta-analysis
Source: BMC Gastroenterol. 2016 Jul 26;16:80. doi: 10.1186/s12876-016-0491-7 (PMC4962503; doi:10.1186/s12876-016-0491-7)
Supplement: Additional file 3: Table S1. — Excluded studies based on full-text review. (DOCX 14 kb) [file 12876_2016_491_MOESM3_ESM.docx]

**Supplementary Table 1. Excluded studies based on full-text review (n=26)**

(Listed in alphabetical order according to the last name of the first author)

| **Study** | **Title** | **Reason for exclusion** |
| --- | --- | --- |
| Buzas  2003 | Eradication of Helicobacter pylori infection in Hungary (1993-2002): a meta-analysis | Individual studies all prior to 2002 |
| Buzas  2007 | Nitrofuran-based regimens for the eradication of Helicobacter pylori infection | Furan is currently not available in the UK/US market |
| Fakheri  2001 | Clarithromycin vs. furazolidone in quadruple therapy regimens for the treatment of Helicobacter pylori in a population with a high metronidazole resistance rate. | Updated article existed |
| Fischbach  2004 | Meta-analysis: the efficacy, adverse events, and adherence related to first-line anti-Helicobacter pylori quadruple therapies. | Meta-analysis containing observational studies |
| Ford  2003 | How can the current strategies for Helicobacter pylori eradication therapy be improved | Variation of dose or duration for the same drug combination |
| Ford  2008 | Adverse events with bismuth salts for Helicobacter pylori eradication: systematic review and meta-analysis. | No comparison |
| Gatta  2013 | Global eradication rates for Helicobacter pylori infection: Systematic review and meta-analysis of sequential therapy. | Variation of dose or duration for the same drug combination |
| Gisbert  2012 | Review article: rifabutin in the treatment of refractory Helicobacter pylori infection. | Meta-analysis containing observational studies |
| Gisbert  2011 | Review article: the effectiveness of standard triple therapy for Helicobacter pylori has not changed over the last decade, but it is not good enough. | Variation of dose or duration for the same drug combination |
| Gisbert  2011 | Review article: non-bismuth quadruple (concomitant) therapy for eradication of Helicobater pylori. | Not using eradication rate as outcome measure |
| Gong 2014 | Meta-analysis of first-line triple therapy for helicobacter pylori eradication in Korea: is it time to change | No comparison |
| Huang  2010 | The furazolidone-based first-line therapy for Helicobacter Pylori Infection:A Meta-analysis | Furan is currently not available in the UK/US market |
| Jodlowski 2008 | Emerging therapies for the treatment of Helicobacter pylori infections | No meta-analysis |
| Kate  2013 | Sequential therapy versus standard triple-drug therapy for Helicobacter pylori eradication: a systematic review of recent evidence. | Variation of dose or duration for the same drug combination |
| Khurana  2007 | Meta-analysis: Helicobacter pylori eradication treatment efficacy in children. | Special population |
| Kim  2013 | Sequential therapy for helicobacter pylori infection in Korea: Systematic review and meta-analysis. | Variation of dose or duration for the same drug combination |
| Li BZ 2015 | Comparative effectiveness and tolerance of treatments for Helicobacter pylori: systematic review and network meta-analysis | The RCTs in each of the meta-analysis were not clearly specified with article identification characteristics (e.g. author name/reference in forest plots) |
| Marin  2013 | A review of rescue regimens after clarithromycin-containing triple therapy failure (for Helicobacter pylori eradication) | No comparison |
| Rodgers  2007 | A meta-analysis of the success rate of Helicobacter pylori therapy in Canada | Meta-analysis containing observational studies |
| Satoh  2009 | Second-line therapy for patients with Helicobacter pylori eradication failure. [Japanese] | No meta-analysis |
| Segarra-Newnham  2012 | Salvage options for eradication of Helicobacter pylori during tetracycline backorder | No meta-analysis |
| Sierra  2014 | Ideal treatment for Helicobacter pylori: a systematic review. [Spanish] | No meta-analysis |
| Wang 2014 | Standard triple therapy for Helicobacter pylori infection in China: A meta-analysis | Not comparing specific pharmacological regimens |
| Zhu  2014 | Levofloxacin based triple therapy for eradication of Helicobacter pylori: a meta-analysis. [Chinese] | Very poor quality: not any characteristics details and reference of the included studies at all. |
| Zullo  2012 | Furazolidone-based therapies for Helicobacter pylori infection: a pooled-data analysis. | Meta-analysis containing observational studies |
| Zullo  2013 | Modified sequential therapy regimens for Helicobacter pylori eradication: A systematic review. | Variation of dose or duration for the same drug combination |
