# Supplementary material for: Pharmacological regimens for eradication of Helicobacter pylori: an overview of systematic reviews and network meta-analysis
Source: BMC Gastroenterol. 2016 Jul 26;16:80. doi: 10.1186/s12876-016-0491-7 (PMC4962503; doi:10.1186/s12876-016-0491-7)
Supplement: Additional file 4: Table S2. — Quality assessment of included studies based on revised AMSTAR checklist. (DOCX 17 kb) [file 12876_2016_491_MOESM4_ESM.docx]

**Supplementary Table 2. Quality assessment of included studies based on revised AMSTAR checklist (n=30)**

| Categories | First author, publication year | 1.1  Clearly stated research question | 1.2 Selection& extraction by two people | 1.3  At least two major database searched | 1.4  Clearly specified selection criteria | 1.5  List of included/ excluded studies | 1.6  Clearly extracted study characteristics | 1.7  Quality assessed &documented | 1.8  Quality used appropriately | 1.9  Result appropriately combined | 1.10 publication bias assessed | 1.11 conflict of interest declared | **Overall*** |
| --- | --- | --- | --- | --- | --- | --- | --- | --- | --- | --- | --- | --- | --- |
| different PPIs | Gisbert 2003-r | √ | √ | √ | √ | × | √ | ⃝ | ⃝ | √ | × | √ | **+** |
|  | Vergara 2003 | √ | √ | × | √ | √ | × | √ | × | √ | × | √ | **+** |
|  | Gisbert 2004-p | √ | √ | √ | √ | ⃝ | √ | √ | × | √ | ⃝ | √ | **+** |
|  | Gisbert 2004 -E | √ | ⃝ | √ | √ | ⃝ | √ | ⃝ | √ | √ | ⃝ | √ | **+** |
|  | Wang X 2006 | √ | √ | √ | √ | × | √ | √ | ⃝ | √ | √ | × | **0** |
|  | Wang ZH 2006 | √ | √ | √ | × | × | √ | √ | × | √ | × | × | **0** |
|  | McNicholl 2012 | √ | √ | √ | ⃝ | √ | ⃝ | × | × | √ | √ | √ | **0** |
| different antibiotics | Zhang ZF 2008 | √ | ⃝ | √ | × | × | × | √ | √ | √ | √ | × | **0** |
|  | Dong 2009 | √ | √ | √ | √ | √ | √ | √ | ⃝ | √ | √ | × | **+** |
|  | Yuan 2009 | √ | √ | √ | ⃝ | √ | √ | √ | ⃝ | ⃝ | ⃝ | √ | **+** |
|  | Zhang 2013 | √ | √ | √ | √ | √ | √ | √ | √ | √ | ⃝ | √ | **+** |
|  | Ye 2014 | √ | √ | √ | √ | √ | √ | √ | ⃝ | √ | √ | √ | **++** |
|  | Peedikayil 2014 | √ | √ | √ | × | √ | √ | √ | √ | √ | √ | √ | **+** |
|  | Xiao 2014 | √ | √ | √ | √ | √ | √ | √ | √ | √ | √ | √ | **++** |
|  | Gou 2014 | √ | √ | ⃝ | × | × | × | × | × | √ | √ | ⃝ | **0** |
| PPI based triple vs. bismuth based therapy | Gene 2003 | √ | ⃝ | × | × | √ | √ | ⃝ | × | √ | ⃝ | √ | **0** |
|  | Gisbert 2005 | √ | √ | √ | √ | × | √ | √ | √ | √ | × | √ | **+** |
|  | Saad 2006 | √ | √ | √ | √ | ⃝ | √ | × | × | × | ⃝ | × | **0** |
|  | Gisbert 2006 | √ | √ | √ | ⃝ | ⃝ | √ | √ | × | ⃝ | × | √ | **0** |
|  | Luther 2010 | √ | √ | √ | × | √ | √ | × | × | √ | √ | √ | **0** |
|  | Li 2010 | √ | √ | √ | √ | √ | √ | × | × | × | ⃝ | √ | **0** |
|  | Wu 2011 | ⃝ | √ | √ | √ | √ | ⃝ | √ | √ | √ | √ | √ | **++** |
|  | Di Caro 2012 | √ | √ | √ | √ | × | ⃝ | × | × | √ | × | × | **0** |
|  | Venerito 2013 | √ | √ | √ | √ | √ | √ | × | × | √ | √ | √ | **+** |
| PPI vs. H_2_RA | Graham 2003 | √ | √ | √ | √ | × | ⃝ | × | × | √ | × | √ | **0** |
|  | Gisbert 2003 | √ | ⃝ | √ | √ | √ | √ | ⃝ | ⃝ | √ | × | √ | **+** |
|  | Ren 2010 | √ | √ | √ | ⃝ | √ | √ | √ | √ | √ | ⃝ | × | **++** |
| Others | Gisbert 2012 | × | × | √ | √ | × | √ | × | × | ⃝ | × | √ | **0** |
|  | Lv 2015 | √ | √ | √ | √ | √ | √ | √ | ⃝ | √ | √ | × | **+** |
|  | Nishizawa 2014 | √ | √ | √ | √ | √ | √ | √ | ⃝ | ⃝ | √ | × | **+** |

* Quality assessment: High quality (++): Majority of criteria met. Little or no risk of bias. Results unlikely to be changed by further research. Acceptable (+): Most criteria met. Some flaws in the study with an associated risk of bias, Conclusions may change in the light of further studies. Low quality (0): Either most criteria not met, or significant flaws relating to key aspects of study design. Conclusions likely to change in the light of further studies

Individual items in the checklist: “Yes”: √; “No”: ×; “Can’t say”: ⃝
