# Supplementary material for: Pharmacological regimens for eradication of Helicobacter pylori: an overview of systematic reviews and network meta-analysis
Source: BMC Gastroenterol. 2016 Jul 26;16:80. doi: 10.1186/s12876-016-0491-7 (PMC4962503; doi:10.1186/s12876-016-0491-7)
Supplement: Additional file 5: Table S3. — Individual study check table. (DOCX 52 kb) [file 12876_2016_491_MOESM5_ESM.docx]

**Supplementary Table 3. Individual study check table**

**Supplementary Table 3-1. Triple therapy with different PPIs**

| Individual study (first author, year) | Gisbert 2004--Esome | Gisbert 2003 --Rabe | Gisbert 2004--Panto | Wang X 2006 | Wang ZH 2006 | McNicholl 2012 | Vergara 2003 |
| --- | --- | --- | --- | --- | --- | --- | --- |
| Miehlke 2003 | √ |  |  | √ | √ | √ |  |
| Tulassay 2001 | √ |  |  | √ | √ |  | √ |
| Van Zanten 2000 | √ |  |  | √ | √ |  | √ |
| Van Zanten 2003 | √ |  |  | √ | √ |  |  |
| Dojo 2001 |  | √ |  |  |  | √ | √ |
| Inaba 2002 |  | √ |  |  |  | √ | √ |
| Kawabata 2001 |  | √ |  |  |  |  |  |
| Kawai 2001 |  | √ |  |  |  |  |  |
| Kuwayama 2001 |  | √ |  |  |  | √ |  |
| Miki 2001 |  | √ |  |  |  |  |  |
| Miwa 1999 |  | √ |  |  |  | √ | √ |
| Miwa 2000 |  | √ |  |  |  | √ | √ |
| Miyoshi 2001 |  | √ |  |  |  |  |  |
| Murakami 2002 |  | √ |  |  |  | √ | √ |
| Vakil 2002 |  | √ |  |  |  | √ | √ |
| Wong 2001 |  | √ |  |  |  | √ |  |
| Adamek 1997 |  |  | √ |  |  |  |  |
| Cammarota 1999 |  |  | √ |  |  |  |  |
| Catalano 1999 |  |  | √ |  |  |  |  |
| Catalano 2000 |  |  | √ |  |  |  |  |
| Dominguez-Martin 1998 |  |  | √ |  |  |  |  |
| Rinaldi 1999 |  |  | √ |  |  |  |  |
| Salces 2001 |  |  | √ |  |  |  |  |
| Hsu 2005 |  |  |  | √ |  |  |  |
| Sheu 2005 |  |  |  | √ | √ | √ |  |
| Chen 2005 |  |  |  | √ | √ |  |  |
| Anagnostopoulos 2004 |  |  |  | √ | √ |  |  |
| Maev 2003 |  |  |  | √ |  | √ |  |
| Zhang 2004 |  |  |  | √ | √ |  |  |
| Xie 2005 |  |  |  | √ | √ |  |  |
| Zuo 2005 |  |  |  |  | √ |  |  |
| Liu 2006 |  |  |  |  | √ |  |  |
| Adachi 2003 |  |  |  |  |  | √ |  |
| Catalano 2002 |  |  |  |  |  | √ |  |
| Choi 2007 |  |  |  |  |  | √ |  |
| De los Rios 2009 |  |  |  |  |  | √ |  |
| Fernandez-bermejo 2001 |  |  |  |  |  | √ |  |
| Hawkey 2003 |  |  |  |  |  | √ |  |
| Huh 2004 |  |  |  |  |  | √ |  |
| Kang 2008 |  |  |  |  |  | √ |  |
| kawabata 2003 |  |  |  |  |  | √ |  |
| kawai 2007 |  |  |  |  |  | √ |  |
| Kim 2003 |  |  |  |  |  | √ |  |
| Kositchaiwat 2003 |  |  |  |  |  | √ |  |
| Kumar 2007 |  |  |  |  |  | √ |  |
| Lee 2010 |  |  |  |  |  | √ |  |
| Miki 2003 |  |  |  |  |  | √ |  |
| Pan 2010 |  |  |  |  |  | √ |  |
| Subei 2007 |  |  |  |  |  | √ |  |
| Wu 2007 |  |  |  |  |  | √ |  |
| Yang 2003 |  |  |  |  |  | √ |  |
| Zhang 2010 |  |  |  |  |  | √ |  |
| Misiewicz 1997 |  |  |  |  |  |  | √ |
| Spinzi 1998 |  |  |  |  |  |  | √ |
| Catalano 1997 |  |  |  |  |  |  | √ |
| Aydin 1998 |  |  |  |  |  |  | √ |
| Kositchaiwat 2002 |  |  |  |  |  |  | √ |

**Supplementary Table 3-2. Triple therapy with different antibiotics**

| Individual study (first author, year) | Dong 2009 | Yuan 2009 | Zhang 2008 | Zhang  2013 | Xiao 2014 | Gou 2014 | Ye  2014 | Peedikayil 2014 |
| --- | --- | --- | --- | --- | --- | --- | --- | --- |
| Cammarota 1996 | √ |  |  |  |  |  |  |  |
| Caselli 1997 | √ |  |  |  |  |  |  |  |
| Chen 2002 | √ |  |  |  |  |  |  |  |
| Chen 2004 | √ |  |  |  |  |  |  |  |
| Iacopini 2005 | √ |  | √ |  | √ |  | √ | √ |
| Ivashkin 2002 | √ |  |  |  |  |  |  |  |
| Kang 2006 | √ |  |  |  |  |  |  |  |
| Laine 1999 | √ |  |  |  |  |  |  |  |
| Laurent 2001 | √ |  |  |  |  |  |  |  |
| Leri 1997 | √ |  |  |  |  |  |  |  |
| Lu 2007 | √ |  |  |  |  |  |  |  |
| Trevisani 1998 | √ |  |  |  |  |  |  |  |
| Vcev 2000 | √ |  |  |  |  |  |  |  |
| Zhao 2005 | √ |  |  |  |  |  |  |  |
| Nista 2005 |  | √ |  | √ |  |  |  |  |
| Bago 2007 |  | √ |  | √ |  |  |  |  |
| Liu 2007 |  | √ |  |  |  |  |  |  |
| Kilic 2008 |  | √ |  | √ |  |  |  |  |
| Shu 2006 |  |  | √ |  |  |  |  |  |
| Jin 2007 |  |  | √ |  |  |  |  |  |
| Cao 2007 |  |  | √ |  |  |  |  |  |
| Zhang 2008 |  |  | √ |  |  |  |  |  |
| Rispo 2007 |  |  | √ |  |  |  |  |  |
| Nista 2006 |  |  | √ |  | √ |  | √ | √ |
| Gong 2007 |  |  | √ |  |  |  |  |  |
| Zeng 2007 |  |  | √ |  |  |  |  |  |
| Luo 2008 |  |  | √ |  |  |  |  |  |
| Gao 2007 |  |  | √ |  |  |  |  |  |
| Cheon 2006 |  |  |  | √ |  |  |  |  |
| Kang 2007 |  |  |  | √ |  |  |  |  |
| Bago 2009 |  |  |  | √ |  |  |  |  |
| Zheng 2010 |  |  |  | √ |  |  |  |  |
| Molina-Infante 2010 |  |  |  |  | √ |  | √ |  |
| Choi 2011 |  |  |  |  | √ |  |  | √ |
| Liou 2010 |  |  |  |  | √ |  | √ | √ |
| Assem 2010 |  |  |  |  | √ |  | √ | √ |
| Cuadrado-Lavin 2012 |  |  |  |  | √ |  | √ |  |
| Hung 2009 |  |  |  |  | √ |  | √ | √ |
| Chen 2010 |  |  |  |  | √ |  | √ | √ |
| Fu 2010 |  |  |  |  |  | √ |  |  |
| Liu YH 2009 |  |  |  |  |  | √ |  |  |
| Liu ZY 2009 |  |  |  |  |  | √ |  |  |
| Wu 2009 |  |  |  |  |  | √ |  |  |
| Zuo 2010 |  |  |  |  |  | √ |  |  |
| Zhang HB 2012 |  |  |  |  |  | √ |  |  |
| Zhang YM 2011 |  |  |  |  |  | √ |  |  |
| Cheng 2010 |  |  |  |  |  | √ | √ |  |
| Li YC 2012 |  |  |  |  |  | √ |  |  |
| Shu QW 2007 |  |  |  |  |  | √ |  |  |
| Wang JZ 2011 |  |  |  |  |  | √ |  |  |
| Wang ZR 2011 |  |  |  |  |  | √ |  |  |
| Wang CY 2012 |  |  |  |  |  | √ |  |  |
| Qi 2013 |  |  |  |  |  | √ |  |  |
| Luo 2010 |  |  |  |  |  | √ |  |  |
| Hu L 2008 |  |  |  |  |  | √ |  |  |
| Hu JH 2008 |  |  |  |  |  | √ |  |  |
| Fan 2013 |  |  |  |  |  | √ |  |  |
| Rong 2009 |  |  |  |  |  | √ |  |  |
| Guo 2009 |  |  |  |  |  | √ |  |  |
| Ma 2008 |  |  |  |  |  | √ |  |  |
| Antos 2006 |  |  |  |  |  |  | √ |  |

**Supplementary Table 3-3. Triple therapy versus bismuth-based therapy**

| Individual study (first author, year) | Gene 2003 | Saad 2006 | Gisbert 2005 | Wu 2011 | Li 2010 | Luther 2010 | Gisbert 2006 | Di Caro  2012 | Venerito  2013 |
| --- | --- | --- | --- | --- | --- | --- | --- | --- | --- |
| Calvet 2002 | √ |  |  |  |  | √ |  |  | √ |
| Gomollon 2000 | √ |  |  |  |  | √ |  |  | √ |
| Laine 2000 | √ |  |  |  |  |  |  |  |  |
| Katelaris 1999 | √ |  |  |  |  |  |  |  |  |
| Nista 2003 |  | √ |  |  | √ |  | √ | √ |  |
| Perri 2003 |  | √ |  |  | √ |  | √ | √ |  |
| Bilardi 2004 |  | √ |  |  | √ |  | √ | √ |  |
| Wong 2004 |  | √ |  |  | √ |  |  | √ |  |
| Nista 2004 |  | √ |  |  |  |  |  | √ |  |
| Nista 2005 |  | √ |  |  | √ |  | √ | √ |  |
| Watanabe 2003 |  | √ |  |  |  |  |  |  |  |
| Zullo 2003 |  | √ |  |  |  |  |  |  |  |
| Cammarota 2004 |  | √ |  |  |  |  |  |  |  |
| Gatta 2004 |  | √ |  |  |  |  |  |  |  |
| Festa 2002 |  | √ |  |  |  |  |  |  |  |
| Bago 2002 |  |  | √ |  |  |  |  |  |  |
| Bujanda 2001 |  |  | √ |  |  |  |  |  |  |
| Bujanda 2001-2 |  |  | √ |  |  |  |  |  |  |
| Cabooter 1999 |  |  | √ |  |  |  |  |  |  |
| Catalano 1998 |  |  | √ |  |  |  |  |  |  |
| Georgopoulos 1999 |  |  | √ |  |  |  |  |  |  |
| Gisbert 2000 |  |  | √ |  |  |  |  |  |  |
| Ravizza 1998 |  |  | √ |  |  |  |  |  |  |
| Rojas 2000 |  |  | √ |  |  |  |  |  |  |
| Salces 2000 |  |  | √ |  |  |  |  |  |  |
| Spinzi 2000 |  |  | √ |  |  |  |  |  |  |
| Sung 1998 |  |  | √ |  |  |  |  |  |  |
| Susi 1999 |  |  | √ |  |  |  |  |  |  |
| Triossi 1998 |  |  | √ |  |  |  |  |  |  |
| Danese 2001 |  |  | √ |  |  |  |  |  |  |
| Delle Cave 1998 |  |  | √ |  |  |  |  |  |  |
| Farup 2002 |  |  | √ |  |  |  |  |  |  |
| Geccherle 1999 |  |  | √ |  |  |  |  |  |  |
| Peyre 1998 |  |  | √ |  |  |  |  |  |  |
| Savarino 2000 |  |  | √ |  |  |  |  |  |  |
| Spadaccini 1998 |  |  | √ |  |  |  |  |  |  |
| Van't Hoff 1999 |  |  | √ |  |  |  |  |  |  |
| Wong 2001 |  |  | √ |  |  |  |  |  |  |
| Bardhan 2001 |  |  | √ |  |  |  |  |  |  |
| Chuang 2001 |  |  | √ |  |  |  |  |  |  |
| Hung 2002 |  |  | √ |  |  |  |  |  |  |
| Cheon 2006 |  |  |  | √ | √ |  |  |  |  |
| Kang 2007 |  |  |  | √ | √ |  |  |  |  |
| Wu 2008 |  |  |  | √ |  |  |  |  |  |
| Bago 2009 |  |  |  | √ | √ |  |  |  |  |
| Dai 2009 |  |  |  | √ |  |  |  |  |  |
| Fang 2009 |  |  |  | √ |  |  |  |  |  |
| Zheng 2010 |  |  |  | √ |  |  |  |  | √ |
| Kuo 2009 |  |  |  |  | √ |  |  | √ |  |
| Jung 2008 |  |  |  |  | √ |  |  | √ |  |
| Zhang 2008 |  |  |  |  | √ |  |  | √ |  |
| Liang 2007 |  |  |  |  | √ |  |  |  |  |
| Zhang 2007 |  |  |  |  | √ |  |  | √ |  |
| Wong 2006 |  |  |  |  | √ |  |  | √ |  |
| Wong 2003 |  |  |  |  | √ |  |  |  |  |
| Peitz 2002 |  |  |  |  | √ |  |  |  |  |
| Qasim 2005 |  |  |  |  | √ |  |  |  |  |
| Peitz 1998 |  |  |  |  | √ |  |  |  |  |
| Mantzaris 2005 |  |  |  |  | √ |  |  |  |  |
| Katelaris 2002 |  |  |  |  |  | √ |  |  | √ |
| Mantzaris 2002 |  |  |  |  |  | √ |  |  | √ |
| Pai 2003 |  |  |  |  |  | √ |  |  | √ |
| Laine 2003 |  |  |  |  |  | √ |  |  | √ |
| Jang 2005 |  |  |  |  |  | √ |  |  | √ |
| Uygun 2007 |  |  |  |  |  | √ |  |  | √ |
| Ching 2008 |  |  |  |  |  | √ |  |  | √ |
| Gisbert 2005 |  |  |  |  |  |  | √ |  |  |
| Orsi 2003 |  |  |  |  |  |  | √ | √ |  |
| Wong 2002 |  |  |  |  |  |  | √ |  |  |
| Songur 2009 |  |  |  |  |  |  |  |  | √ |
| Malfertheiner 2011 |  |  |  |  |  |  |  |  | √ |
| Gisbert 2008 |  |  |  |  |  |  |  | √ |  |

**Supplementary Table 3-4. PPI versus H2 receptor antagonist (H2RA) in triple therapy**

| Individual study (first author, year) | Graham 2003 | Gisbert 2003 | Ren 2010 |
| --- | --- | --- | --- |
| Graham 2003 | √ |  |  |
| Savarino 1999 | √ | √ |  |
| Gschwantler 1999 | √ |  |  |
| Lazzaroni 1997 | √ | √ |  |
| Kihira 1999 | √ |  |  |
| Spadaccini 1996 | √ | √ |  |
| Grigoriev 1994 | √ |  |  |
| Lamouliatte 1992 | √ |  |  |
| Ell 2011 | √ | √ |  |
| Tham 1996 | √ | √ |  |
| Hsu 2001 | √ | √ |  |
| Adamek 1995 |  | √ |  |
| Borody 1995 |  | √ |  |
| Cataldo 1996 |  | √ |  |
| Grigoriew 1994 |  | √ |  |
| Gashwantler 1999 |  | √ |  |
| Lamouliatte 1991 |  | √ |  |
| Mosca 1995 |  | √ |  |
| Murakami 1999 |  | √ |  |
| Oderda 1999 |  | √ |  |
| Popovic 1997 |  | √ |  |
| Rusznlewski 1997 |  | √ |  |
| Sacca 1996 |  | √ |  |
| Shcherbakov 2001 |  | √ |  |
| Treiber 1997 |  | √ |  |
| Isomoto 2003 |  |  | √ |
| Hagiwara 2007 |  |  | √ |
| Kim 2008 |  |  | √ |

**Supplementary Table 3-5. Other drug therapies**

Table is not reported as there is no overlapping study in this category.
